# Establishment and characterization of immortalized mouse embryonic fibroblasts by SV40 large T antigen

**DOI:** 10.3389/fcell.2026.1936956

**Published:** 2026-10-22

**Authors:** Xin Li, Ting Shao, Lan Xu, Li Zheng, Yue-Lei Chen

**Affiliations:** 1 National Collection of Authenticated Cell Cultures, Center for Excellence in Molecular Cell Science, Chinese Academy of Sciences, Shanghai, China; 2 University of Chinese Academy of Sciences, Beijing, China

**Keywords:** feeder cells, immortalization, mouse embryonic fibroblasts, SV40 large T antigen, tri-lineage differentiation

## Abstract

Mouse embryonic fibroblasts (MEFs), which are typically derived from mouse embryos between embryonic days 12.5 and 13.5, serve as a widely used feeder layer for pluripotent stem cell culture. MEFs exhibit a limited proliferative capacity and are typically used within five passages after which they undergo early senescence. However, the repeated preparation of primary MEFs introduces batch-to-batch variability and raises ethical concerns regarding animal use, thereby limiting the reproducibility and scalability of stem cell culture. To overcome these limitations, here we established a nontumorigenic immortalized MEF line (designated as SV40-CMEF) from C57BL/6J mice via SV40 large T antigen (SV40LT) transfection. SV40-CMEFs showed genomic integration, enhanced proliferation, and the capacity to support the self-renewal of R1/E mouse embryonic stem cells (mESC) as feeder cells. They also retained their tri-lineage differentiation potential. Notably, SV40-CMEFs did not form colonies in soft agar or tumors in immunodeficient mice. Transcriptomic profiling revealed substantial global remodeling compared with that in primary MEFs; however, functional gene modules associated with feeder capacity, fibroblast identity, and proliferation were selectively retained, whereas senescence markers were suppressed. This cell line provides a convenient, well-characterized resource for stem cell culture and cell differentiation research.

## 1 Introduction

Mouse embryonic fibroblasts (MEFs) represent a classical primary cell model widely used in stem cell research and basic biology. They are most commonly employed as feeder cells for mouse embryonic stem cells because MEFs can synthesize and secrete a variety of growth factors and cytokines, including fibroblast growth factor 2 (FGF2), bone morphogenetic protein 4 (BMP4), leukaemia inhibitory factor (LIF), activin A, and transforming growth factor β1 (TGF-β1) (Yusuf et al., 2013; Choupani et al., 2022). Through the secretion of these growth factors and cytokines, MEFs establish a niche that is essential for maintaining the self-renewal and pluripotent status of embryonic and induced pluripotent stem cells. MEFs are essentially fibroblasts of mesodermal origin and retain the tri-lineage differentiation potential. Under specific induction conditions, they can differentiate into osteocytes, adipocytes, and chondrocytes (Wang et al., 1993; Saeed et al., 2012). Owing to their unique proliferative capacity, secretory functions, and well-defined molecular phenotype, MEFs play irreplaceable roles in diverse fields, such as stem cell culture, gene function studies, and developmental biology. Primary MEFs exhibit limited proliferative capacity and are typically used within five passages, after which the cells undergo senescence (Ferreira and Hein, 2023). Therefore, establishing a genetically defined, publicly available, and functionally validated immortalized MEF line that retains both feeder-layer capacity and differentiation potential is of considerable importance.

Simian vacuolating virus 40 (SV40) was discovered in 1960, and since then, the introduction of SV40 large T antigen (SV40LT) was widely applied as one of the most effective approaches for establishing immortalized cell lines. Scientists have reported that the SV40 early encoded large T antigen can bind to p53 and retinoblastoma (Rb) proteins and activate telomerase, thereby bypassing the normal replicative limit and leading to cell immortalization (Kim et al., 2001; de Bardet et al., 2023). Although alternative immortalization methods have been developed—most notably hTERT-mediated immortalization, which extends cellular lifespan through telomere maintenance—SV40LT continues to be favored for its high efficiency and the extensive functional characterization of the resulting cell lines (Toouli et al., 2002; de Bardet et al., 2023; Ni et al., 2024).

In this study, we established an SV40LT antigen-immortalized MEF cell line (designated as SV40-CMEF) and performed comprehensive characterization of its biological properties, including persistence of SV40LT, proliferative capacity, fibroblast identity, genetic authentication, tumorigenicity evaluation, and genome-wide transcriptomic profiling. Moreover, we focused specifically on the comprehensive characterization of their stem cell-supporting capacity and tri-lineage differentiation potential. This newly established cell line is expected to provide a valuable cellular resource for studies on stem cell culture, development, and differentiation.

## 2 Materials and methods

### 2.1 Cell culture and establishment of SV40-CMEF

Primary MEFs were isolated from postcoitus day 13.5 embryos obtained from C57BL/6J mice purchased from SLAC Laboratory Animal Co., Ltd. (Cat# 0050895), following established protocols with minor modifications (Ferreira and Hein, 2023; Jozefczuk et al., 2012). The culture medium was prepared with DMEM (Gibco, Cat# 11995065) supplemented with 10% FBS (Gibco, Cat# A5669701) and 1% nonessential amino acids Solution (Gibco, Cat# 11140050).

To establish an immortalized MEF cell line, C57BL/6J MEFs at passage 3 were transfected with the pLenti CMV/TO SV40LT-GFP plasmid using Lipofectamine 3000 reagent (Thermo Fisher, Cat# L3000001) as recommended by the manufacturer. Immortalized cells were obtained through natural selection by continuous passaging without drug selection. The resulting immortalized MEF line was designated SV40-CMEF. The SV40-CMEF line (*Mus musculus*) is publicly accessible through the China Center for Type Culture Collection (CCTCC) and the National Collection of Authenticated Cell Cultures. Under the long-term culture, the SV40-CMEF cells were subjected to *mycoplasma* testing routinely using the *Mycoplasma* Detection Kit (TaKaRa, Cat# CY232). After deposited to cell repository, PCR was re-preformed to ensure that the SV40-CMEF cells were mycoplasma-free.

### 2.2 DNA extraction, PCR and quantitative PCR for copy number of SV40LT

Total DNA was isolated with the QuickExtract™ DNA Extraction Solution 1.0 (LGC Biosearch Technologies, Cat# QE0905T) following the manufacturer’s instructions. The target sequences were amplified with 2 × Rapid Taq Master Mix (Vazyme, Cat# P222-01). The primers used are detailed in Supplementary Table S1. The resulting amplicons were separated on a 2% agarose gel alongside DNA Marker II (TIANGEN, Cat# MD102) for size reference. Briefly, quantitative PCR was performed using SV40LT-specific primers and SYBR® Green Realtime PCR Master Mix (TOYOBO, Cat# QPK-201). Mouse Actb was used to quantify the number of diploid genomes.

### 2.3 SA-β-gal assay

SA-β-Gal activity was assessed with a Senescence β-Galactosidase Staining Kit (Beyotime, Cat# C0602) following the manufacturer’s instructions. In brief, after a 15-min fixation at room temperature, cells were kept overnight at 37 °C in staining solution within a CO_2_-free incubator, after which images were captured with an Olympus CKX53 microscope. All experiments were independently repeated three times.

### 2.4 Total RNA extraction and real-time PCR (RT-PCR)

Total RNA was isolated with the TaKaRa MiniBEST Universal RNA Extraction Kit (TaKaRa Bio, Cat# 9767). Reverse transcription and semi-quantitative PCR were performed in a single step using the One-Step TB Green PrimeScript RT‒PCR Kit II (Perfect Real Time) (TaKaRa Bio, Cat# RR096A), as instructed by the manufacturer. Gene expression levels were measured on a QuantStudio 1 system (Thermo Fisher Scientific). Gene expression levels were determined with the QuantStudio Design and Analysis software using the comparative Ct method, which employs the comparative Ct (ΔΔCt) method with amplification efficiency correction. *Gapdh* served as the endogenous reference gene, and fold changes were determined relative to those in the primary MEF control group. All primer information is provided in Supplementary Table S1. The results were drawn from three separate experiments.

### 2.5 Western blot assay

Cells were collected when confluence surpassed 90%. The cell pellets were lysed in RIPA lysis buffer (Medium) (Beyotime, Cat# P0013C). Proteins were separated on 4%–20% SurePAGE gels (GenScript, Cat# M00657) and transferred to PVDF (Polyvinylidene fluoride) membranes. The membranes were then immersed in primary antibody solution and kept at 4 °C overnight. The antibodies used were anti-GAPDH (Cell Signaling Technology, Cat# 2118), anti-Vimentin (Cell Signaling Technology, Cat# 5741), anti-α-SMA (Cell Signaling Technology, Cat# 19245), anti-SV40 Large T Antigen (Cell Signaling Technology, Cat# 15729), and anti-S100A4 (Proteintech, Cat# 84221-1-RR). Following a 1-h incubation with goat anti-rabbit IgG (Thermo Fisher-Invitrogen, Cat# C31460100) at room temperature, the blots were developed with BeyoECL Moon working solution (Beyotime, Cat# P0018FS), after which the samples were exposed to a gel imaging system.

### 2.6 Cell proliferation assay and crystal violet assay

Cells were plated in 96-well plates (5,000 cells/well) and left undisturbed for 4 h at 37 °C for attachment prior to real-time confluence monitoring. Cell confluence was monitored over time using a PerkinElmer Ensight Microplate Reader (PerkinElmer). For EdU incorporation assay, cells were seeded in 96 well plates (3,000–5000 cells/well). EdU was then added to the culture medium at a final concentration of 10 μM. After incubated for 2 h at 37 °C, cells were fixed, permeabilized and subjected to Click reaction using the EdU Cell Proliferation Kit with AF555 (Beyotime, Cat# C0076S) according to the manufacturer’s instructions. Nuclei were counterstained with Hoechst 33342. Images were captured and quantified using an Opera Phenix Plus High Content Screening System (Revvity). For the crystal violet assay, cells were seeded at 1 × 10^5^ cells per well. At each time point, cells were incubated with Crystal Violet Staining Solution (Beyotime, Cat# C0121) following the manufacturer’s instructions. The incorporated dye was extracted with 36% acetic acid for 10 min, and the absorbance at 595 nm was recorded on a microplate reader.

### 2.7 Immunofluorescence staining assay

Cells were immobilised with 4% paraformaldehyde (Beyotime, Cat# P0099) for 20 min at room temperature, permeabilized with Permeabilization Solution for immunostaining (Proteintech, Cat# PR30007), and blocked with 5% Goat Serum (Beyotime, Cat# C0265) in PBS for 1 h at ambient temperature. The cells were then probed overnight at 4 °C with primary antibodies against Oct-4A (1:200; Cell Signaling Technology, Cat# 2840), Sox2 (1:200; Bioss, Cat# 60788R), Nanog (1:200; Cell Signaling Technology, Cat# 8822), vimentin, α-SMA, and S100A4 (the same antibodies used for Western blotting), followed by incubation with fluorescent secondary antibodies (Proteintech, Cat# RGAR004/RGAR002) for 1 h in the dark. The nuclei were counterstained with DAPI (Proteintech, Cat# PR30021). Images were captured using a fluorescence microscope (Nikon CSU-W1).

### 2.8 Flow cytometry

For cell cycle profiling, cells were immobilized in ice-cold 70% ethanol, maintained at 4 °C for no less than 30 min, rinsed twice with PBS supplemented with 2% FBS, and then exposed to FxCycle™ PI/RNase Staining Solution (Thermo Fisher-Invitrogen, Cat# F10797) for 15–30 min at room temperature with protection from light. Cells were analyzed immediately using PE-Texas Red excitation. For EGFP detection, cell pellets were collected and analyzed immediately using FITC excitation. Flow cytometry was performed on a BD LSR Fortessa™ (BD Biosciences).

### 2.9 Cell short tandem repeat (STR) identification and karyotype analysis

STR analysis was outsourced to Microread Genetics (Beijing, China). Genomic DNA was extracted using the Microread Genomic DNA Kit and amplified with the Mouse STR Genotyping Kit (Microread Genetics, Mouse STR v2.0), targeting 18 mouse STR loci and 2 human loci. PCR products were detected on an ABI 3730xl Genetic Analyzer. STR match rates were calculated using the Tanabe algorithm: Match rate (%) = (Total matched alleles × 2)/(Total alleles in both samples) × 100% (Tanabe et al., 1999). The data were analysed using GeneMapper ID-X software (Applied Biosystems) and compared against the ExPASy database.

For karyotype analysis (performed by Microread Genetics), cells were treated with colcemid prior to harvest, and metaphase spreads were prepared by standard hypotonic/fixation procedures and Giemsa-trypsin banding. Slides were scanned using a GSL-120 automatic scanner, and chromosomes in 100 cells per sample were counted using Cytovision software.

### 2.10 Anchorage-independent assay

Anchorage-independent growth was assessed by soft agar assay, following established protocols (Willmer et al., 2016). In brief, each plate received a base layer of 0.6% agar in culture medium (1:1 mixture of 1.2% agar and medium), followed by a top layer of 0.35% agar containing 2.5 × 10^4^ cells. Plates were incubated in a CO_2_ incubator for 3–4 weeks, stained with Iodonitrotetrazolium Chloride (INT) (Shyuanye, Cat# S19047) stained with 1 mg/mL p-iodonitrotetrazolium chloride at 37 °C for 16–18 h, after which images were captured. All experiments were independently repeated three times.

### 2.11 BALB/c nude mice tumorigenicity assay

BALB/c nude mice, obtained from SLAC Laboratory Animal Co., Ltd. (Cat# 0057288), were used for xenograft experiments. The Lewis lung carcinoma (LLC) cell line served as a positive control. Healthy 4-week-old female BALB/c nude mice received a subcutaneous injection of the cell suspension (acclimated in the barrier facility for >3 days) at 100 µL per mouse (1 × 10^7^ cells/mL, equivalent to 1 × 10^6^ cells). Animals were randomly assigned to two groups (LLC and SV40-CMEF; n = 5 per group) and monitored daily for tumor formation. Tumor size was maintained within institutional ethical limits. If no tumors developed within 1 month, the mice were maintained for up to 3 months. Images were captured at appropriate time points.

### 2.12 Application of immortalized SV40-CMEF as feeder cells

SV40-CMEFs were irradiated to serve as feeder cells. The flasks were coated with 0.5% gelatine at 37 °C for 30 min prior to seeding the feeder cells at an appropriate density. After overnight, once the feeder cells had attached, R1/E mouse embryonic stem cells were plated onto the monolayer. The culture medium for R1/E cells was DMEM containing 10% FBS, 1× GlutaMAX (Gibco, Cat# 35050061), 1% nonessential amino acids Solution, leukaemia inhibitory factor (LIF) (Sigma‒Aldrich, Cat# ESG1107), and 1.5 mM 2-mercaptoethanol (Gibco, Cat# 21985023). For passaging, the differential adhesion method was used: the cells were detached with TrypLE Express Enzyme, incubated at 37 °C for 2–3 min, resuspended, and placed in a new flask. After 1 h, the supernatant containing R1/E cells was collected and reseeded onto a fresh feeder-seeded flask. The colony-forming efficiency was quantified by the procedure as described previously (Qu et al., 2018). Briefly, R1/E cells were seeded onto feeder layers at a density of 50 cells/cm^2^ and cultured for 5 days Colonies were fixed with 4% paraformaldehyde and stained using an alkaline phosphatase (AP) staining kit (Beyotime, Cat# C3250S) according to the manufacturer’s instructions.

### 2.13 Tri-lineage differentiation of SV40-CMEFs

For differentiation assays, plates were pre-coated with gelatin to improve cell attachment. Cells were seeded and cultured until they surpassed 90% confluence. Uninduced control cells were maintained in culture medium. Differentiation media were prepared as follows. Adipogenic differentiation employed two media used in alternating cycles. Medium A: DMEM + 10% FBS + 1 µM dexamethasone (Beyotime, Cat# ST1258) + 500 µM IBMX (Beyotime, Cat# ST1398) + 2 µM rosiglitazone (Solarbio, Cat# SR8600) + 10 μg/mL insulin (Beyotime, Cat# P3378). Medium B: DMEM + 10% FBS + 10 μg/mL insulin. Medium A was applied for 2 days, followed by Medium B for 2 days; this cycle was repeated until differentiation was complete (Zebisch et al., 2012). The cells were exposed to Oil Red O solution (OriCell, Cat# OILR-10001). Osteogenic differentiation medium: DMEM + 10% FBS + 50 μg/mL ascorbic acid (Beyotime, Cat# ST1434) + 5 mM β-glycerophosphate (Macklin, Cat# G997397) + 10 nM dexamethasone (Metzger et al., 2025). The culture medium was replaced at intervals of 2–3 days. Cells were incubated with Alizarin Red S solution (OriCell, Cat# ALIR-10001). Chondrogenic differentiation medium: DMEM (without FBS) + 37.5 μg/mL ascorbic acid + 1 × ITS (Gibco, Cat# 41400045) + 10 nM dexamethasone + 1 × sodium pyruvate (Gibco, Cat# 11360070) + 10 ng/mL TGF-β (Novoprotein, Cat# CA59). The serum-free control group was maintained in DMEM + 1% nonessential amino acids Solution. The culture medium was replaced at 2- to 3-day intervals. At the end of the induction period, cells were immobilized with 4% paraformaldehyde for 15 min, rinsed with DPBS, and then exposed to Alcian blue 8GX solution (OriCell, Cat# ALCB-10001) for 1 h, and rinsed with distilled water. All staining procedures followed the manufacturers’ protocols. Images were captured under a microscope. For semi-quantitative analysis of chondrogenic differentiation as described previously (Yang et al., 2021), parallel stained wells were incubated with 6 M guanidine hydrochloride for 12 h at room temperature and the elusion was then measured at 595 nm using a microplate reader.

### 2.14 RNA-seq

Primary C57BL/6J MEFs were used as the control group, and SV40-CMEFs as the experimental group. The cell pellets were collected and immediately frozen in liquid nitrogen. High-throughput RNA sequencing was performed at GENEWIZ (Suzhou, China) using the Illumina platform. Following isolation, total RNA integrity and purity were verified via agarose gel electrophoresis, Nanodrop spectrophotometry, Qubit fluorometry, and capillary electrophoresis. Polyadenylated transcripts were then captured with oligo (dT)-coated magnetic beads, fragmented, and reverse-transcribed into double-stranded cDNA. Libraries were constructed through the standard Illumina workflow—including end-repair, A-tailing, adapter ligation, size selection, and PCR enrichment—and, after final quality control, paired-end sequencing (2 × 150 bp) was carried out on an Illumina platform. The data were obtained from three independent experiments. Raw reads were processed with Fastp (v0.24.1) and assessed with FastQC (v0.11.9). Clean reads were aligned to the mouse reference genome (GRCm38/mm10) using HISAT2 (v2.2.1) with Ensembl annotations. Gene-level counts were obtained with HTSeq (v0.6.1). Differential expression analysis was performed with DESeq2 (v1.34.0). Counts were normalized using the median-of-ratios method, and p values were adjusted with the Benjamini–Hochberg procedure. Genes with |log2FoldChange| ≥ 1 and false discovery rate (FDR) < 0.05 were considered differentially expressed. Principal component analysis (PCA) was performed on fragments per kilobase of transcript per million mapped reads (FPKM) values using the R function prcomp. Volcano plots and a heatmap of 35 selected genes across six functional categories were generated. Kyoto Encyclopedia of Genes and Genomes (KEGG) pathway enrichment analysis of differentially expressed genes was performed using a hypergeometric test, with adjusted p value (Q value) < 0.05 considered significantly enriched. Gene Set Enrichment Analysis (GSEA) was performed using gseapy (v1.1.9) against Gene Ontology (GO) biological process and KEGG collections. Normalized enrichment scores (NES) and false discovery rates (FDR) were reported. For GSEA input, normalized counts per million (CPM) were obtained using edgeR, and genes with CPM > 1 in at least 50% of samples were retained. The signal-to-noise metric was used for ranking, with 1,000 permutations, and gene sets with FDR < 0.25 were considered significantly enriched. GO and KEGG annotations were retrieved from org.Mm.eg.db.

### 2.15 Image analysis and statistics

The results of the Western blot, immunofluorescence, and image analyses were quantified using Fiji/ImageJ (version 2.18.0/1.54p; National Institutes of Health, Bethesda, MD, USA). Statistical analyses were performed using Excel (version 2019) or GraphPad Prism (version 10). For experiments requiring multiple comparisons, statistical significance was assessed by ANOVA, data are presented as mean ± SD (n = 3). *P < 0.05, **P < 0.01, ***P < 0.001. Principal component analysis (PCA) was performed and visualized using R statistical software (version 4.5.0). Volcano plots of differentially expressed genes were generated using R.

## 3 Results

### 3.1 Integration and expression of the SV40 large T antigen in SV40-CMEFs

Initially, primary MEFs were derived from the mouse C57BL/6J strain, which has a clear genetic background and growth characteristics, making them an ideal cell model for the present work. An immortalized cell pool was then established from the MEFs via the transfection of nonviral SV40LT transfection to preserve the functional diversity of the parental MEFs. Primary MEFs typically undergo replicative senescence within five passages and were therefore used without passage-level subclassification. For SV40-CMEFs, two passage windows were defined to capture potential changes associated with long-term culture: low passage (LP, ≤25 passages) and high passage (HP, ≥75 passages).

PCR analysis using primers specific for the SV40LT gene confirmed the presence of the transgene in SV40-CMEFs (Figure 1A). Semi-quantitative PCR analysis further confirmed comparable *SV40LT* signals in SV40-MEFs (Figure 1B). Western blot analysis confirmed the expression of the SV40LT at the protein level, with a single specific band detected at the expected molecular weight (Figure 1C). The SV40-expressing plasmid also carried an EGFP reporter cassette, allowing the transfection efficiency to be directly assessed by flow cytometry. EGFP fluorescence was detected in 95% of the cells within the established cells (Figure 1D), confirming highly efficient delivery and sustained expression of the transgene. Subsequently, the copy number of *SV40LT* was determined by standard-curve qPCR using *SV40LT*-specific primers (Supplementary Figure S1A). As presented in Supplementary Figure S1B, the *SV40LT* copy number was approximately 4 copies per genome in SV40-CMEFs at LP and 5 copies per genome in SV40-CMEFs at HP, indicating that *SV40LT* integrates within the host genome. Moreover, a ∼3.4 kb length of DNA fragment carrying the full-length SV40LT-IRES2-EGFP cassette was specifically amplified from the genomic DNA of SV40-CMEFs (Supplementary Figure S1C), suggesting physical linkage between the transgene and the reporter in the polyclonal SV40-CMEF pool. Together, these results demonstrate that the SV40LT gene was persistently expressed and genomic integrated in SV40-CMEFs across extended passages, irrespective of passage number.

**FIGURE 1:**
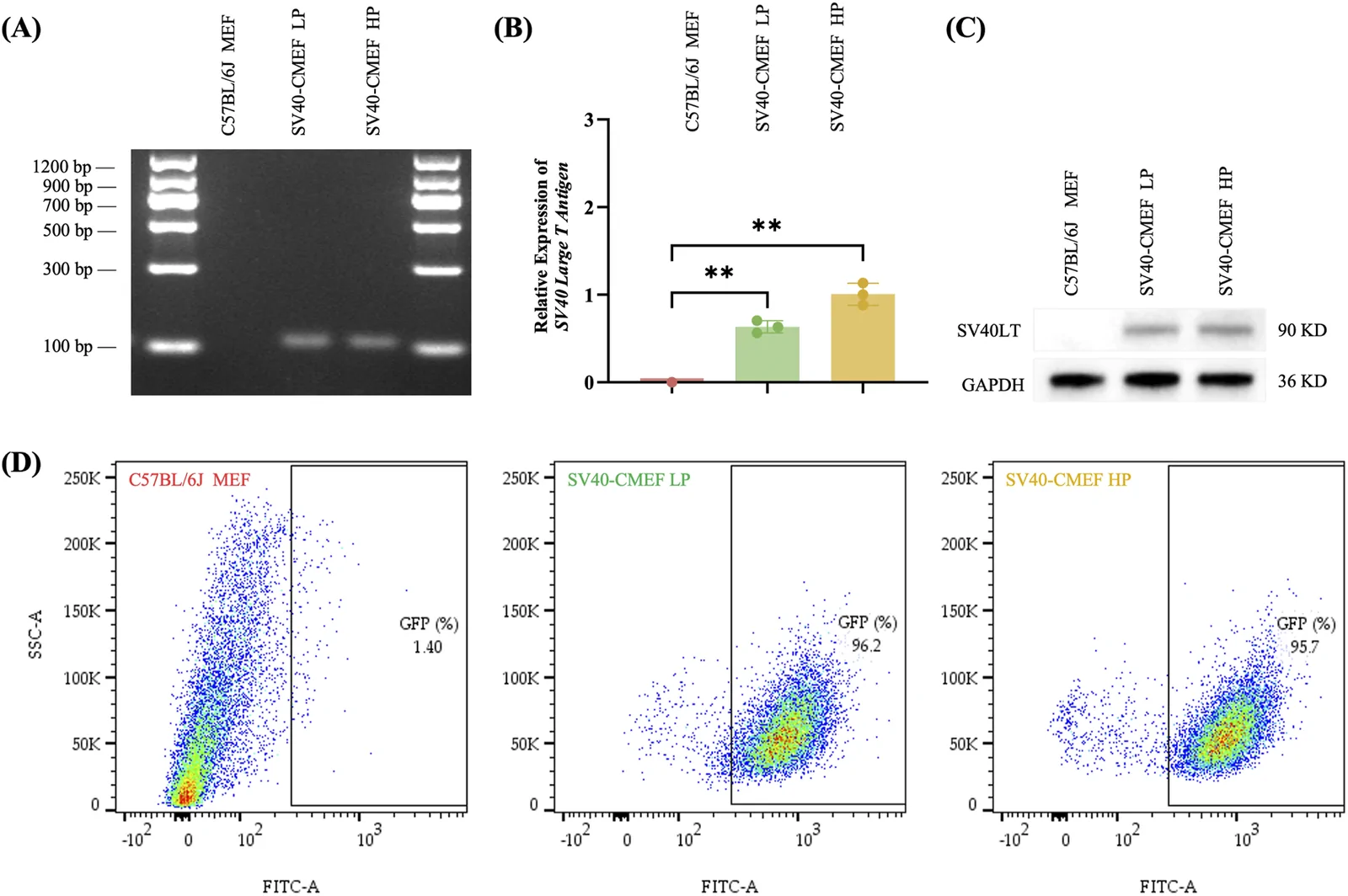
The SV40LT transgene is maintained and expressed across passages in SV40-CMEFs. For SV40-CMEFs, two passage windows were defined to capture potential changes as-sociated with long-term culture: low passage (LP, ≤25 passages) and high passage (HP, ≥75 passages). **(A)** PCR validation of SV40LT genomic integration in SV40-CMEFs at low passage (LP, ≤ P25) and high passage (HP, ≥ P75). **(B)** Semi-quantitative PCR (qPCR) confirming the genomic integration of the transgene. **(C)** Western blot analysis of the protein expression of the SV40LT in primary MEFs and SV40-CMEFs. **(D)** Flow cytometric analysis of EGFP expression showing high transfection efficiency (>95%) in the established SV40-CMEF.

### 3.2 SV40-CMEFs exhibit escape senescence, and acquire enhanced proliferative capacity

Primary MEFs displayed a heterogeneous, spindle-shaped fibroblast-like morphology, whereas SV40-CMEFs at both LP and HP exhibited a more uniform morphology (Figure 2A). Senescence-associated β-galactosidase (SA-β-gal) staining confirmed that primary MEFs at late passages exhibited typical senescence-associated blue staining, as indicated by arrows, whereas this senescent phenotype was absent SV40-CMEFs (Figure 2B). To quantitatively assess the proliferative capacity of SV40-CMEFs at both LP and HP, real-time cell confluence was monitored using a live-cell imaging microplate system. SV40-CMEFs exhibited a robust and continuous proliferation pattern over time, in striking contrast to the slower growth observed in parental primary MEFs (Figure 2C). This enhanced proliferation was further validated by a crystal violet assay, which revealed a progressive increase in cell density at different time points, as confirmed by the absorbance data (Figure 2D). Furthermore, flow cytometric analysis of DNA content was performed to evaluate cell-cycle distribution. Given the extensive aneuploidy in SV40-CMEFs (Figure 3), standard cell-cycle fitting was not applicable; instead, DNA content histograms were generated after doublet discrimination. The resulting profiles showed shifted DNA-content distributions in SV40-CMEFs compared with primary MEFs (Figure 2E). To provide an orthogonal measure of cell-cycle progression, an EdU incorporation assay was employed to monitor DNA replication in primary MEFs and SV40-CMEFs. The quantification data of EdU incorporation support the observation on accelerated cell-cycle progression in SV40-CMEFs (Figures 2F,G). Together, these data demonstrate that SV40-CMEFs escape replicative senescence and acquire increased proliferative capacity.

**FIGURE 2:**
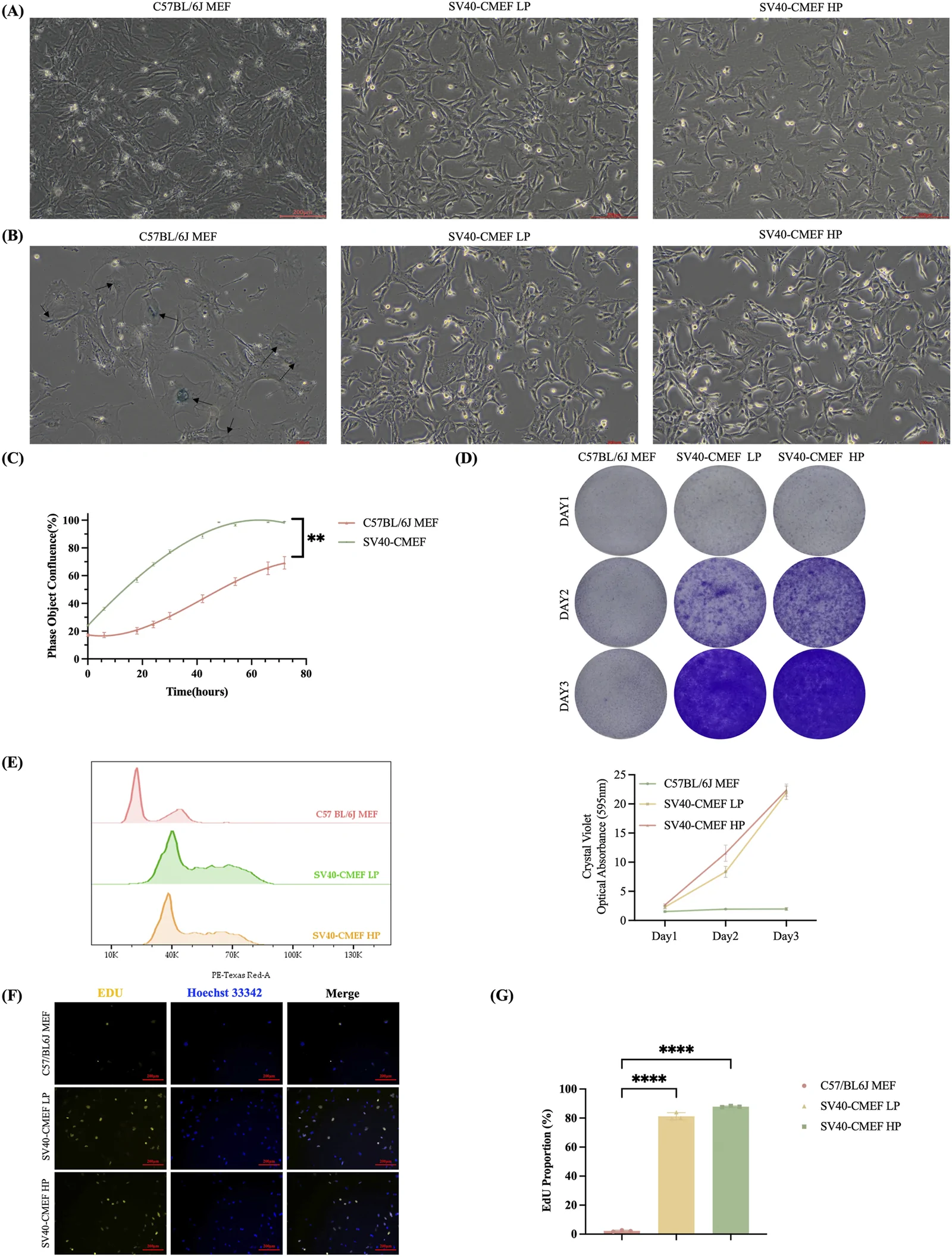
Morphological characterization and proliferation analysis of SV40-CMEFs. **(A)** Representative phase-contrast images of primary MEFs at early passage (P1), SV40-CMEFs at both LP and HP. Scale bar, 200 μm. **(B)** SA-β-gal staining demonstrating senescence in primary MEFs at late passage (P4) (blue staining, marked by arrows) and the absence of staining in different passage of SV40-CMEFs. Scale bar, 200 μm. **(C)** Real-time proliferation curves monitored by an Ensight microplate reader, showing changes in cell confluence over time. **(D)** Crystal violet staining assay. Upper: representative images of stained cells at different time points. Lower: quantification of cell proliferation by measuring the absorbance at 595 nm after dye elution with 33% acetic acid. **(E)** Based on the flow cytometric analysis of DNA content in primary MEFs and SV40-CMEFs as indicated, DNA content histograms are shown. **(F)** Representative EdU fluorescence images of primary MEFs at late passage (P4) and SV40-CMEFs as indicated. EdU-positive nuclei are shown in yellow, and nuclei were counterstained with Hoechst 33342 (blue). Scale bar, 200 μm. **(G)** Quantification of EdU-positive cells in primary MEFs and SV40-CMEFs.

**FIGURE 3:**
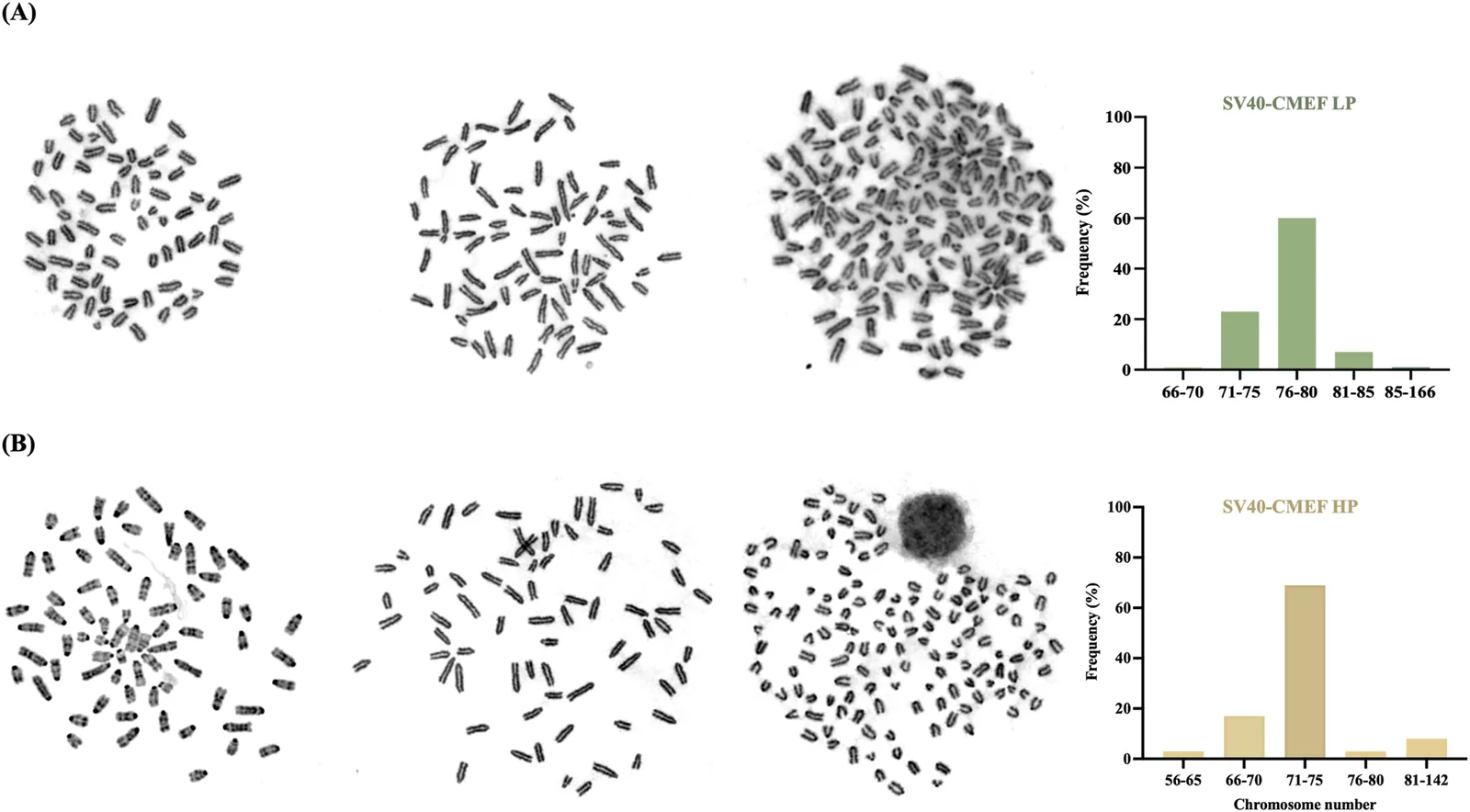
Karyotype analysis of SV40-CMEFs showing aneuploidy. **(A)** Chromosome numbers of SV40-CMEF at LP ranged from 68 to 166. Among the 100 metaphase spreads counted, the modal chromosome number was 78. **(B)** Chromosome numbers of SV40-CMEF at HP ranged from 59 to 142. Among the 100 metaphase spreads counted, the modal chromosome number was 72.

### 3.3 Characterization of fibroblast markers in immortalized SV40-CMEFs

To investigate whether the fibroblast phenotype was affected by SV40LT-mediated immortalization, the expression and localization of key fibroblast markers were examined. Immunofluorescence staining and quantitative analysis revealed that primary MEFs and SV40-CMEFs displayed characteristic filamentous vimentin staining and cytosolic expression of α-SMA (α-smooth muscle actin) and S100A4, confirming their fibroblast identity (Figure 4A). The transcript levels of these markers were quantified by semi-qPCR. Consistent with the immunofluorescence data, the mRNA expression of *Vimentin*, *α-SMA*, and *S100A4* in SV40-CMEFs at both LP and HP mirrored the protein-level trends, confirming the retention of fibroblast identity at the transcriptional level (Figure 4B). Western blot analysis further confirmed these trends at the protein level, and densitometric quantification of band intensities corroborated both the immunofluorescence and RT-PCR results (Figure 4C). Collectively, these findings demonstrate that SV40-CMEFs retain fibroblast identity, as evidenced by the persistent production of Vimentin, α-SMA, and S100A4 at the protein level, the persistent expression of the corresponding genes at the transcript level, and the retention of their cellular localization.

**FIGURE 4:**
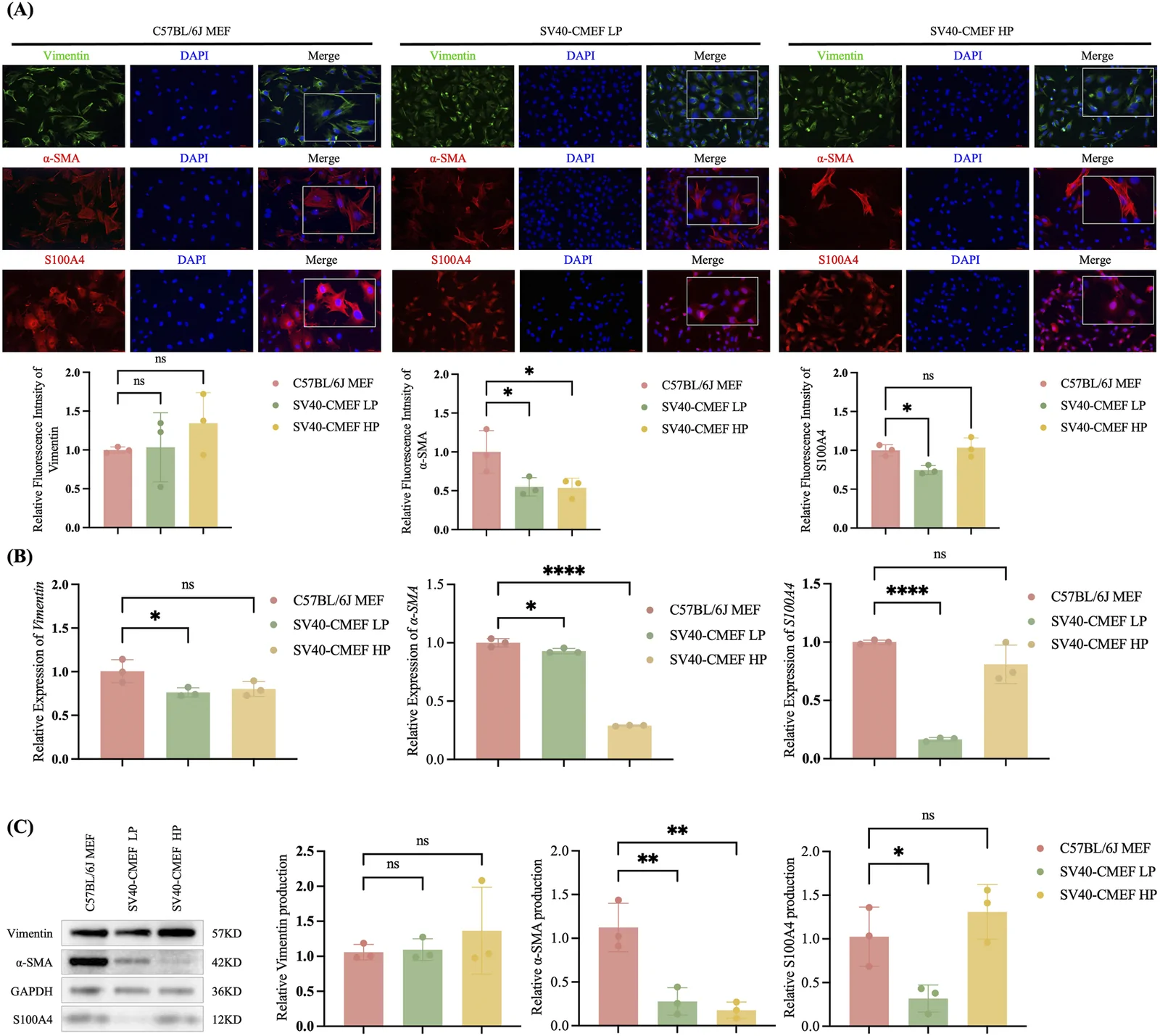
SV40-CMEFs retain fibroblast identity. **(A)** Immunofluorescence staining and quantification of vimentin, α-SMA, and S100A4 expression in primary MEFs and SV40-CMEFs. DAPI staining (blue) labelled the nuclei. The bar graphs show the mean fluorescence intensity ±SD. Scale bar, 100 μm. **(B)** Semi-qPCR analysis of *vimentin*, *α-SMA*, and *S100A4* mRNA expression. The data are normalized to those of *Gapdh* and are presented as the mean ± SD (n = 3). **(C)** Western blot analysis and densitometric quantification of Vimentin, α-SMA, and S100A4 expression at the protein level. GAPDH served as the loading control. The bar graphs show the relative protein expression normalized to that of GAPDH.

### 3.4 SV40-CMEFs retain cellular identity and exhibit karyotypic alterations

Subsequently, short tandem repeat (STR) analysis was performed to confirm the origin and genetic identity of the established cell line. The STR profiles of SV40-CMEFs showed an extremely high similarity with those of the parental primary C57BL/6J MEFs (Supplementary Table S3). These results confirm that the SV40-CMEFs were derived from the intended C57BL/6J strain MEFs without cross-contamination. Meanwhile, karyotype analysis was performed to determine whether SV40-mediated immortalization induced the chromosomal abnormalities. In contrast to the normal diploid karyotype (2n = 40) of primary MEFs, SV40-CMEFs displayed extensive aneuploidy. After counting 100 metaphase spreads, chromosome numbers of LP cells ranged from 68 to 166 per metaphase spread, and the modal chromosome number was 78 (Figure 3A). Similarly, chromosome numbers of HP cells ranged from 59 to 142 per metaphase spread with a modal chromosome number of 72 (Figure 3B), implying a gradual reduction in chromosome numbers across passages (Supplementary Table S3). These findings indicate that SV40LT-induced immortalization results in substantial chromosomal numerical instability in MEFs.

### 3.5 SV40-CMEFs are nontumorigenic in vitro and in vivo

The tumorigenic potential of SV40-CMEFs was assessed using both anchor-age-independent growth and *in vivo* tumor formation assays. LLC cells, a C57BL/6-derived Lewis lung carcinoma line, were used as a positive control. In a soft agar colony formation assay, LLC cells readily formed colonies, whereas primary MEFs and SV40-CMEFs showed no such capacity (Figure 5A). These results indicate that SV40-CMEFs do not possess the capacity for anchorage-independent growth. SV40-CMEFs and LLC cells were subcutaneously injected into BALB/c nude mice for further *in vivo* tumorigenicity assessment. LLC cells formed palpable tumors within 2–3 days and reached the ethically permitted size limit within approximately 2 weeks, at which point the tumors were excised. In contrast, no tumor formation was observed in mice injected with SV40-CMEFs throughout the 3-month observation period (Figure 5B). These findings demonstrate that SV40-CMEFs do not acquire tumorigenic potential *in vivo*, which is consistent with the soft agar results.

**FIGURE 5:**
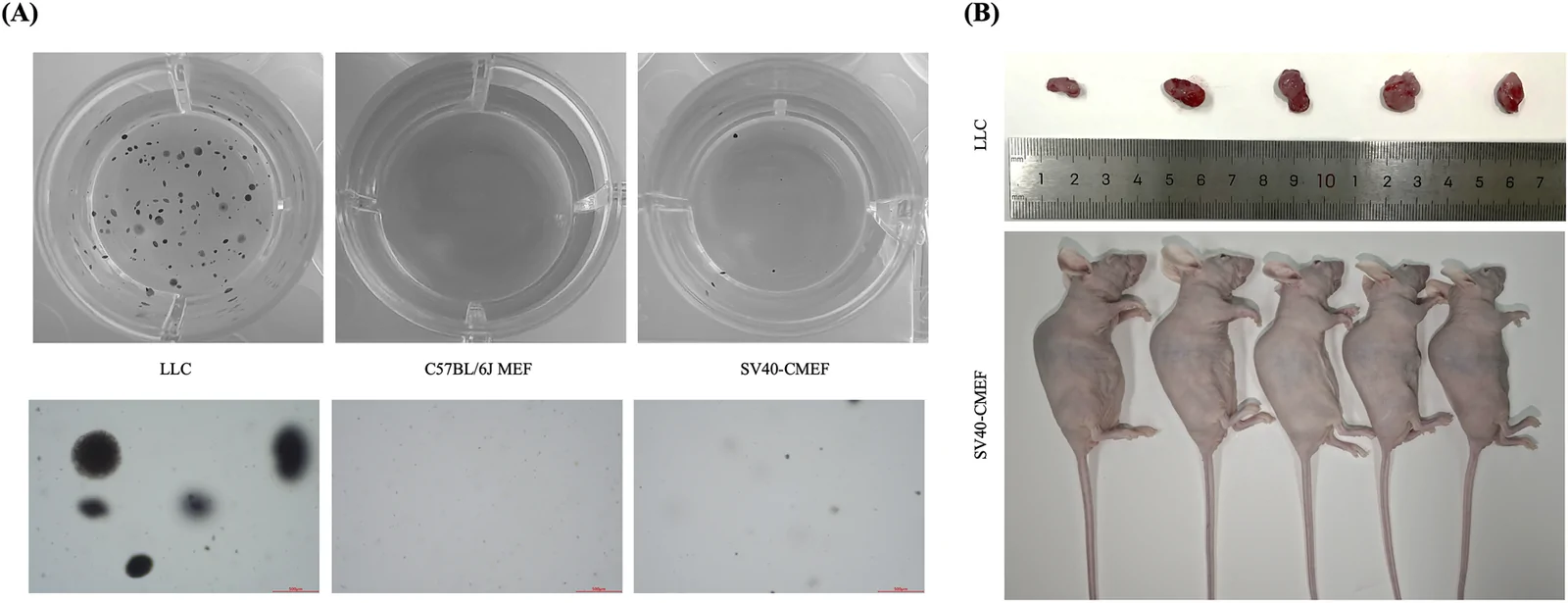
SV40-CMEFs lack tumorigenic potential both *in vitro* and *in vivo*. **(A)** Soft agar colony formation assay. Upper panel: macroscopic overview of wells seeded with primary MEFs, SV40-CMEFs, and LLC cells (positive control). Lower panel: representative microscopy images of the same wells, showing colony formation only in LLC cells. Scale bar, 500 μm. **(B)** *In vivo* tumorigenicity assay in BALB/c nude mice. Upper panel: tumors excised from LLC-injected mice 2 weeks after inoculation. Lower panel: Representative mice 3 months after subcutaneous injection of SV40-CMEFs, showing no tumor formation.

### 3.6 SV40-CMEFs retain feeder-layer function and tri-lineage differentiation potential

For the evaluation of feeder-layer capacity, SV40-CMEFs were assessed for their ability to support mouse embryonic stem cell culture. As shown in Figure 6A, mouse embryonic stem cell R1/E colonies maintained on SV40-CMEF feeders retained a compact, dome-shaped morphology and were positive for the pluripotency marker Oct-4A, Sox2 and Nanog as detected by immunofluorescence. Meanwhile, we analyzed the colony-forming efficiency and proportion of AP-positive undifferentiated colonies that were similar between primary MEF and SV40-CMEF feeders, with no statistically significant difference (Supplementary Figure S2). These results demonstrate that SV40-CMEFs support self-renewal of mouse embryonic stem cells. These results demonstrate that immortalized SV40-CMEFs can sustain mouse embryonic stem cells in an undifferentiated state without compromising their pluripotency.

**FIGURE 6:**
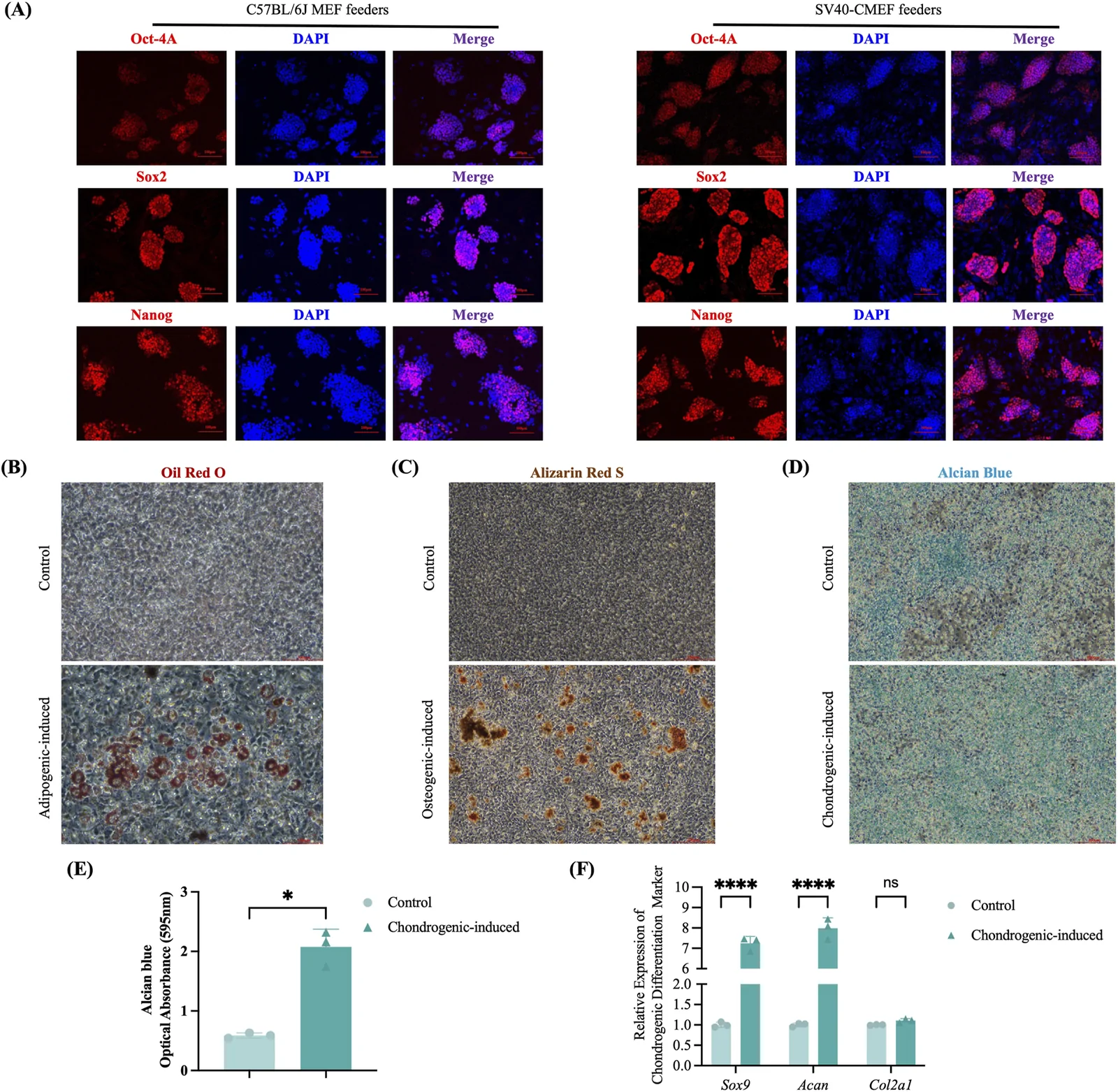
Functional characterization of SV40-CMEFs as feeder cells and their multi-lineage differentiation potential. **(A)** Irradiated SV40-CMEFs supporting the growth of R1/E mouse embryonic stem cells. After cultured for six passages, immunofluorescence staining was applied to detect the expression of Oct4A, Sox2 and Nanog (red) in R1/E colonies. Nuclei were counterstained with DAPI (blue). **(B)** Adipogenic differentiation: Oil Red O staining of SV40-CMEFs after 9 days of induction. Up: uninduced control cells cultured in culture medium, showing no positive staining. Down: induced cells showing strong positive staining with visible lipid droplets. **(C)** Osteogenic differentiation: Alizarin Red S staining of SV40-CMEFs after 7 days of induction. Up: uninduced control, showing no staining. Down: induced cells confirming mineralized matrix deposition. **(D)** Chondrogenic differentiation: Alcian blue staining of SV40-CMEFs after 14 days of induction under serum-free conditions. Up: control cells maintained in serum-free basal medium. Down: induced cells. Both groups showed positive staining; the induced group exhibited more extensive staining, indicating that enhanced proteoglycan synthesis was driven by chondrogenic induction factors. **(E)** Semi-quantification of Alcian blue staining by dye extraction and absorbance measurement in the serum-free control and chondrogenic induced groups. **(F)** RT-PCR analysis of *Sox9*, *Acan* and *Col2a1*, in the serum-free control and chondrogenic induced groups. The bar graphs show relative protein levels normalized to *Gapdh*.

Because the tri-lineage differentiation capacity of primary MEFs has been well documented (Saeed et al., 2012), we focused our assessment on SV40-immortalized cells to determine whether this potential was retained after transformation. SV40-CMEFs were subjected to adipogenic, osteogenic, and chondrogenic induction. For adipogenic and osteogenic differentiation, uninduced cells maintained in basal culture medium served as the control. In terms of adipogenic differentiation, Oil Red O staining was strongly positive after 9 days of induction, confirming the presence of lipid droplets and successful differentiation into mature adipocytes, whereas control cells showed no positive staining (Figure 6B). With respect to osteogenic differentiation, Alizarin Red S staining was positive after 7 days of induction, confirming the deposition of mineralized matrix and osteogenic differentiation, with no staining observed in control cells (Figure 6C). For chondrogenic differentiation, SV40-CMEF was induced under serum-free conditions. The control group was maintained in serum-free basal medium. Both the induced and control groups showed positive Alcian blue staining, confirming the accumulation of proteoglycan (Figure 6D). The staining in the induced group was more extensive than that in the control group, suggesting that the serum-free environment itself promoted a basal level of proteoglycan synthesis, while the chondrogenic induction factors further enhanced this process. To strengthen its specificity as evidence of chondrogenesis, the staining intensity of Alcian blue was then measured by absorbance. The relative staining intensity was significantly higher in the induced group than that in the serum-free control group (Figure 6E). These results were further supported by our detection on the expression of cartilage anabolic genes. As shown in Figure 6F, the expression of *Sox9* and *Acan* were significantly upregulated in the induced group, whereas *Col2a1* mRNA showed a trend toward increased expression but did not reach statistical significance at the examined time point. Collectively, these results demonstrate that SV40-CMEFs retain feeder-layer function and trilineage differentiation potential, confirming the preservation of the core functional properties of primary MEFs.

### 3.7 SV40-CMEFs undergo profound transcriptomic remodeling with selective retention of functional gene modules

Eventually, the genome-wide transcriptional alterations induced by SV40-mediated immortalization were characterized by RNA-seq. Principal component analysis (PCA) revealed that primary MEFs and SV40-CMEFs were distinctly separated along PC1 (79.7% variance), indicating a profound global transcriptomic shift driven by SV40LT (Figure 7A). SV40-CMEFs clustered more tightly than primary MEFs in the three-dimensional PCA plot, suggesting reduced transcriptional heterogeneity upon immortalization. Volcano plot analysis identified thousands of differentially expressed genes (DEGs) between primary MEFs and SV40-CMEFs (Figure 7B). To gain mechanistic insight, we summarized the differentially expressed genes between the two cells (Supplementary Table S4) and subsequently profiled 35 curated genes spanning six functional categories including the fibroblast markers, ECM and adhesion, feeder-layer factors and downstream signaling, differentiation regulators, senescence and cell cycle genes, and metabolic enzymes (Figure 7C). Despite the extensive transcriptomic remodeling, several feeder-layer-associated factors remained detectable or upregulated in SV40-CMEFs, including *Il6st* (GP130) and the coordinately upregulated *Bmp4–Smad1/Smad5* axis, while the upregulation of differentiation-associated transcription factors, including *Runx2*, *Pparg*, and *Twist1*, provided molecular associations for the retained trilineage differentiation capacity. The remaining curated genes were consistent with the transcriptional remodeling expected of immortalized fibroblasts, while the expression of core functional transcription factors further supported the retention of fibroblast identity and differentiation capacity observed. KEGG pathway enrichment analysis revealed that the most prominently enriched pathways were predominantly associated with environmental information processing, cellular processes, genetic information processing, human diseases, and organismal systems (Figure 7D). Pathways related to proliferation and survival, including PI3K–Akt, MAPK, and Ras signaling pathways, were among the most significantly enriched, consistent with the enhanced proliferative capacity of SV40-CMEFs. Notably, the signaling pathway regulating pluripotency of stem cells was significantly enriched, and several upregulated genes within this pathway, such as the Wnt ligand *Wnt7a*, the downstream transcription factor *Tcf7*, and the TGF-β/Activin receptor *Acvr2b* play essential roles in maintaining stem cell self-renewal, providing a possible molecular explanation for the feeder-layer function retained by SV40-CMEFs. In addition, GSEA using KEGG analysis and GO biological process was performed for functional analysis on all gene sets (Supplementary Table S5). As expected, most of the enriched pathways were involved in cell proliferation, including cell cycle, DNA replication, mitotic cytokinesis, which is in good accordance with enhanced proliferative capacity of SV40-CMEFs (Figure 2). Collectively, these findings demonstrate that, despite profound transcriptional reprogramming, the functional modules supporting feeder-layer capacity and differentiation are selectively retained in SV40-CMEFs.

**FIGURE 7:**
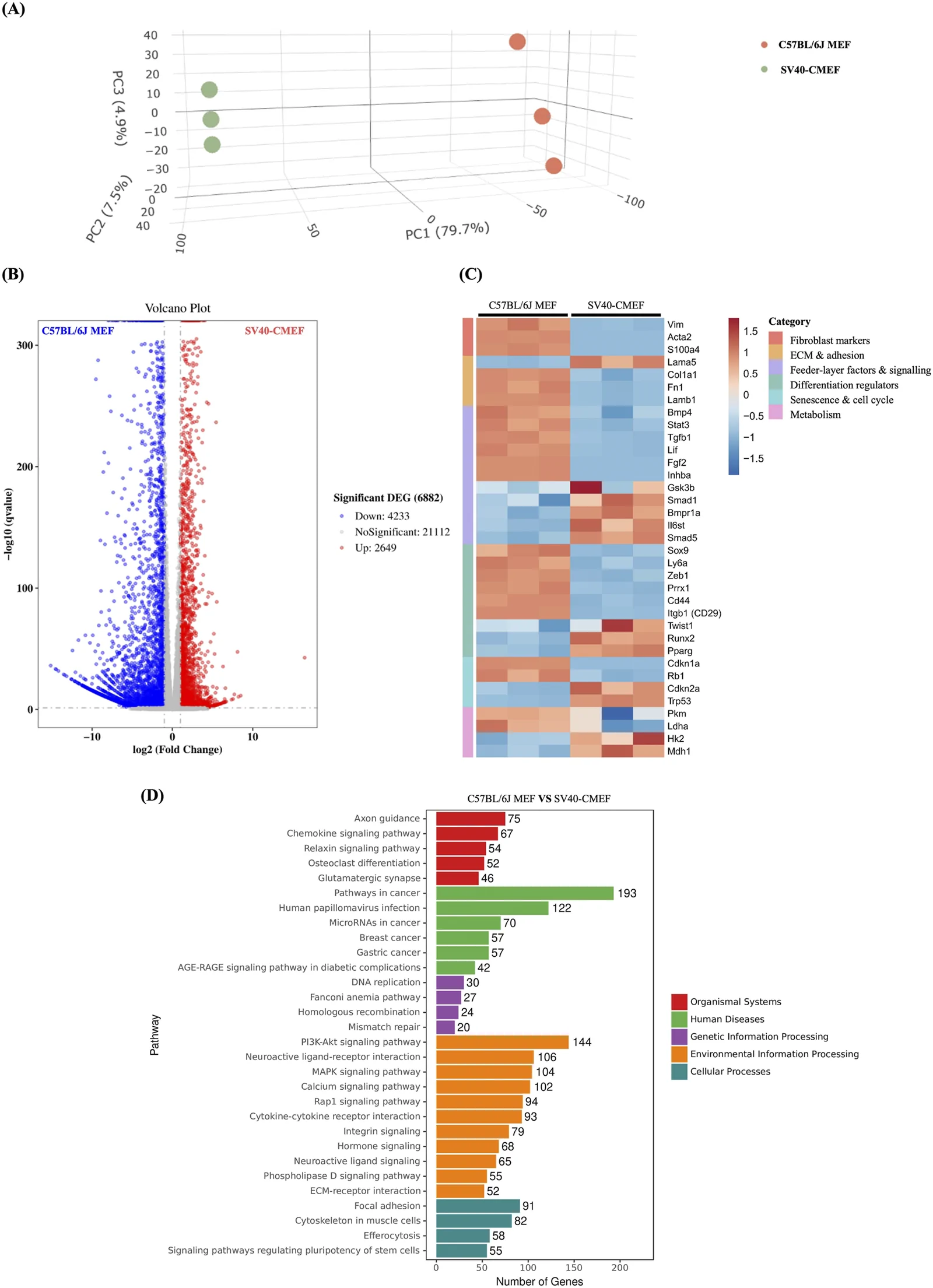
Transcriptomic analysis of SV40-CMEFs. **(A)** Three-dimensional PCA score plot of RNA-seq data from primary MEFs and SV40-CMEFs. PC1 (79.7% variance) clearly separates the two groups, while the tighter clustering of SV40-CMEFs reflects reduced transcriptional heterogeneity. **(B)** Volcano plot of differentially expressed genes (DEGs) between primary MEFs and SV40-CMEFs. **(C)** Heatmap of 35 curated genes across all samples. Gene expression is shown as Z-scores of normalized FPKM values. Genes are grouped by functional category. **(D)** KEGG pathway enrichment analysis of DEGs between primary MEFs and SV40-CMEFs. Pathways are ranked by gene count; the top significantly enriched pathways (Q value <0.05) are shown. Colors indicate KEGG functional categories.

## 4 Discussion

MEFs have long served as a cornerstone in stem cell biology, providing both a feeder-layer substrate for pluripotent stem cell culture and an accessible model for investigating differentiation commitment (Saeed et al., 2012; Yusuf et al., 2013). However, the inherent replicative senescence of primary MEFs—typically occurring within five passages—poses a fundamental limitation to their long-term utility, experimental reproducibility, and scalability. Early demonstrations that SV40 transforms mouse embryo cultures into established lines with unlimited proliferative potential established the foundation for this approach (Kim et al., 2001). Among the various gene delivery methods available, plasmid-based transfection offers practical advantages including its nonviral nature, low-copy genomic integration, and technical simplicity, making it a convenient choice for generating immortalized cell lines without the biosafety concerns associated with viral vectors. In this study, we first established an immortalized MEF line (SV40-CMEF) with feeder-layer function and tri-lineage differentiation potential confirmed by transcriptomic profiling, providing a well-characterized cell model for investigating the molecular basis of feeder-layer support and tri-lineage differentiation commitment.

The genomic integration and sustained expression of the SV40LT transgene across widely spaced passages (Figures 1A–D) recapitulate a key feature of successfully immortalized MEFs, as previously demonstrated using the piggyBac transposon system (Wang et al., 2014). Notably, the plasmid-based method we employed herein achieves transgene retention without auxiliary factors, thereby simplifying the experimental workflow and eliminating potential confounding effects associated with exogenous enzymatic activity. Similarly, the enhanced proliferation and escape from senescence observed in SV40-CMEFs (Figures 2B–F) recapitulate the well-established capacity of SV40LT to bypass p53-and Rb-dependent senescence checkpoints (Kim et al., 2001). STR profiling confirmed the C57BL/6J origin of SV40-CMEFs with a high match rate, verifying that the immortalized cells were derived from the original primary MEFs rather than arising from cross-contamination. This authentication step is critical for all newly established cell lines (Tanabe et al., 1999). In addition, karyotype analysis revealed extensive aneuploidy in SV40-CMEFs (Figure 3). While this degree of karyotypic instability may appear striking, it is in fact consistent with the well-documented tendency of SV40LT to induce chromosomal numerical instability specifically in immortalized fibroblasts. A systematic survey of the literature indicates that SV40-mediated immortalization frequently results in karyotypic abnormalities in fibroblast-derived cells, whereas stable karyotypes are more commonly observed in certain epithelial cell types, likely reflecting cell-type-specific differences in DNA repair capacity and genome maintenance mechanisms (Toouli et al., 2002). Our observations are therefore aligned with the expected behavior of SV40-immortalized fibroblasts, rather than representing an anomaly. Critically, despite this extensive aneuploidy, SV40-CMEFs failed to form colonies in soft agar or tumors in immunodeficient mice (Figures 5A,B), reinforcing the principle that aneuploidy in itself is insufficient for malignant transformation, and that specific oncogenic mutations are required for full neoplastic conversion (Toouli et al., 2002; Fauth et al., 2004).

Functionally, SV40-CMEFs retained the capacity to serve as feeder cells to support R1/E mouse embryonic stem cell self-renewal with maintained the expression of stemness markers (Figure 6A). The concept of using immortalized MEFs as feeder layers was previously validated by Choo et al., who demonstrated that an HPV16 E6/E7-immortalized MEF line could support human embryonic stem cell growth (Choo et al., 2006). Our study extends this concept to an SV40LT-immortalized MEF model, demonstrating that SV40-immortalized MEFs retain feeder-layer function for mouse embryonic stem cell culture. Beyond feeder-layer function, SV40-CMEFs maintained tri-lineage differentiation potential (Wang et al., 1993; Saeed et al., 2012), a property previously demonstrated in SV40-immortalized cell models (Wang et al., 2014). Transcriptomic profiling revealed profound global transcriptomic remodeling relative to primary MEFs (Figures 7A–C). Despite this remodeling, key feeder-layer-associated factors were retained or upregulated in SV40-CMEFs, and differentiation-associated transcription factors remained expressed, providing possible molecular correlates for the retained feeder-layer capacity and differentiation potential. KEGG pathway enrichment analysis further showed that proliferation-related pathways were among the most significantly enriched, consistent with the enhanced proliferative capacity of SV40-CMEFs. In addition, the signaling pathway regulating pluripotency of stem cells was enriched, corroborating the retained feeder-layer function at the transcriptional level (Figure 7D). This pattern of transcriptomic divergence between immortalized and parental cells is not unique to our model; rather, it has been increasingly recognized as a common consequence of cellular immortalization across diverse methods and cell types (Bikkul et al., 2019; Li et al., 2024; Merrick et al., 2025). Collectively, these transcriptomic data corroborate the functional findings that the core properties of primary MEFs, including feeder-layer capacity and differentiation potential, are selectively retained in SV40-CMEFs.

The SV40-CMEF cell line we established provides a genetically defined, publicly available resource for the stem cell and differentiation biology communities. Its demonstrated feeder-layer capacity—a trait known to be influenced by genetic background, cell passage, and isolation timing (Choupani et al., 2022)—eliminates the need for repeated primary MEF preparations, thereby reducing animal usage and improving experimental reproducibility. Previous studies have established that embryonic fibroblasts represent a connecting link between mesenchymal cells and embryonic stem cells (Yusuf et al., 2013) and that MEFs exhibit a phenotype similar, but not identical, to bone marrow stromal stem cells (Saeed et al., 2012). Building upon these foundational observations, the SV40-CMEF model, with its capacity for indefinite expansion and genetic manipulation, could facilitate functional genomics screens aimed at identifying novel regulators of adipogenic, osteogenic, and chondrogenic differentiation. Such screens would leverage established differentiation protocols (Zebisch et al., 2012; Metzger et al., 2025) and the well-documented role of factors such as BMP-2 in mesenchymal lineage commitment (Wang et al., 1993). Indeed, SV40LT-mediated immortalization has been shown to reprogram mouse cardiomyocyte progenitors into cells with mesenchymal stem cell-like properties (Yu et al., 2023), demonstrating that the functional repertoire of immortalized cells can be expanded rather than simply preserved. The non-tumorigenic phenotype of SV40-CMEFs, despite their extensive aneuploidy, positions them as a valuable model for investigating the boundaries between immortalization and malignant transformation. As the field continues to evolve, with various strategies for cell immortalization being explored (de Bardet et al., 2023), the comprehensive characterization workflow employed here—spanning immortalization confirmation, identity authentication, safety evaluation, and functional validation—may serve as a practical reference for validating newly established immortalized cell lines (Huang et al., 2012; Wang et al., 2014). Future studies may explore the utility of SV40-CMEFs in supporting human pluripotent stem cell culture (Jozefczuk et al., 2012), and their potential as a standardized differentiation progenitor model for drug screening and toxicology applications, where a consistent and well-characterized cellular substrate is essential for reproducible results (Willmer et al., 2016).

However, several limitations inherent in this study warrant mention. Firstly, although the SV40-CMEFs have already reached over 300 population doublings after cultured for more than 105 passages, more detailed investigations on the underlying mechanism including p53 inactivation, telomeres length and telomerase activity will be performed to address whether the cells we obtained are truly immortalized. Secondly, cytogenetic aberrations and chromosome instability are recurrent in SV40-immortalized cells. Prior to use, it therefore leads to necessity of further analysis of SV40-CMEFs on structural chromosomal abnormalities using approaches such as FISH, SKY or sequencing-based structural variant analysis. Finally, as the SV40-CMEFs are highly heterogenic, it would differ in the biological characteristics of subclones, such as the integration loci of SV40LT, cell proliferative lifespan and the capacity to support the self-renewal of mESCs. To solve this possible drawback, we are starting to isolate single-cell clones by limiting dilution in the future work.

In summary, SV40-CMEF provides a publicly available, genetically defined resource for stem cell and tri-lineage differentiation research, and the systematic characterization strategy employed here may serve as a reference for validating newly established immortalized cell lines.

## Data Availability

The sequence data have been deposited in the Sequence Read Archive (SRA) database at the NCBI under accession number PRJNA1541626.
