# Supplementary material for: Establishment and characterization of immortalized mouse embryonic fibroblasts by SV40 large T antigen
Source: Front Cell Dev Biol. 2026 Oct 22;14:1936956. doi: 10.3389/fcell.2026.1936956 (PMC13736106; doi:10.3389/fcell.2026.1936956)
Supplement: Supplementary file 1 [file Supplementaryfile1.zip › Supplementary file 1.docx]

Supplementary Material

# 1 Supplementary Figures

# Figure S1. SV40LT integration and cassette structure in SV40-CMEFs. (A) Schematic representation of the SV40LT-IRES2-EGFP cassette, including the SV40LT, IRES2, and EGFP elements and the primer positions used for junction PCR. (B) Standard-curve qPCR quantification of SV40LT copy number per diploid genome in primary C57BL/6J MEFs and SV40-CMEFs as indicated. Data are shown as mean ± SD (n = 3). (C) Using PCR across the SV40LT-EGFP junction, a ~3.4 kb length of DNA fragment carrying the full-length SV40LT-IRES2-EGFP cassette was specifically amplified from the genomic DNA of SV40-CMEFs and then sequenced.

# Figure S2. Colony-forming efficiency and undifferentiated colony quantification. (A) Representative images of AP staining of mESC R1/E colonies in 12-well plates cultured on primary MEF feeders and SV40-CMEF feeders. (B) Colony-forming efficiency of R1/E cells cultured on primary MEF feeders and SV40-CMEF feeders. (C) Proportion of AP-positive undifferentiated colonies on primary MEFs and SV40-CMEFs.

# 2 Supplementary Tables

# Table S1. Primers for PCR and RT-PCR.

| Primer Name | Sequences (5′-3′) |
| --- | --- |
| *SV40LT* Forward | TTCCCTGACCTGAAGGCAAATC |
| *SV40LT* Reverse | GGCTGAACTTTGAGCTAGGAGTAG |
| *SV40 LT* (Junction) Forward | GAGGCTTCTGGGATGCAACT |
| EGFP (Junction) Reverse | CTCGATGTTGTGGCGGATCT |
| Mouse *Gapdh* Forward | AGGTCGGTGTGAACGGATTTG |
| Mouse *Gapdh* Reverse | TGTAGACCATGTAGTTGAGGTCA |
| Mouse *ACTB* Forward | GGCTGTATTCCCCTCCATCG |
| Mouse *ACTB* Reverse | CCAGTTGGTAACAATGCCATGT |
| Mouse *Vimentin* Forward | TGAGATCGCCACCTACAGGA |
| Mouse *Vimentin* Reverse | TTGCGCTCCTGAAAAACTGC |
| Mouse α*-SMA* Forward | GTCCCAGACATCAGGGAGTAA |
| Mouse α*-SMA* Reverse | TCGGATACTTCAGCGTCAGGA |
| Mouse *S100A4* Forward | TCCTCTCTCTTGGTCTGGTCT |
| Mouse *S100A4* Reverse | CAACAGGGAAGATCCCCCAC |
| Mouse *Sox9* Forward | AGCGAACGCACATCAAGAC |
| Mouse *Sox9* Reverse | CTGTAGGCGATCTGTTGGGG |
| Mouse *Acan* Forward | CCCGGTACCCTACAGAGACA |
| Mouse *Acan* Reverse | ACAGTGACCCTGGAACTTGG |
| Mouse *Col2a1* Forward | TGGACGCCATGAAGGTTTTCT |
| Mouse *Col2a1* Reverse | TGGGAGCCAGATTGTCATCTC |

# Table S2. STR analysis of SV40-CMEFs.

| STR Loci | C57BL/6J MEF | | SV40-CMEF | |
| --- | --- | --- | --- | --- |
| 1-1 | 15 | 16 | 16 | 16 |
| 1-2 | 19 | 19 | 19 | 19 |
| 2-1 | 15 | 15 | 15 | 15 |
| 3-2 | 14 | 14 | 14 | 14 |
| 4-2 | 20.3 | 20.3 | 20.3 | 20.3 |
| 5-5 | 17 | 18 | 18 | 18 |
| 6-4 | 18 | 18 | 18 | 18 |
| 6-7 | 17 | 17 | 17 | 17 |
| 7-1 | 27.2 | 27.2 | 27.2 | 27.2 |
| 8-1 | 16 | 16 | 16 | 16 |
| 11-2 | 16 | 16 | 16 | 16 |
| 12-1 | 17 | 17 | 17 | 17 |
| 13-1 | 17.1 | 17.1 | 17.1 | 17.1 |
| 15-3 | 22.3 | 22.3 | 22.3 | 22.3 |
| 17-2 | 16 | 17 | 16 | 17 |
| 18-3 | 16 | 16 | 16 | 16 |
| 19-2 | 13 | 13 | 13 | 13 |
| X-1 | 27 | 28 | 27 | 28 |
| Match Rate | 97.22% | | | |

# Table S3. Chromosome number distribution across 100 metaphase spreads of SV40-CMEFs.

| SV40-CMEF LP | | SV40-CMEF HP | |
| --- | --- | --- | --- |
| Chromosome Number | Number of cells | Chromosome Number | Number of cells |
| 68 | 1 | 59 | 1 |
| 71 | 5 | 64 | 1 |
| 72 | 3 | 65 | 1 |
| 73 | 5 | 66 | 2 |
| 74 | 8 | 68 | 7 |
| 75 | 10 | 69 | 2 |
| 76 | 11 | 70 | 6 |
| 77 | 10 | 71 | 10 |
| 78 | 16 | 72 | 21 |
| 79 | 12 | 73 | 10 |
| 80 | 11 | 74 | 17 |
| 81 | 4 | 75 | 11 |
| 82 | 2 | 77 | 1 |
| 83 | 1 | 78 | 1 |
| 166 | 1 | 80 | 1 |
|  |  | 85 | 1 |
|  |  | 86 | 1 |
|  |  | 87 | 1 |
|  |  | 94 | 1 |
|  |  | 103 | 1 |
|  |  | 110 | 1 |
|  |  | 138 | 1 |
|  |  | 142 | 1 |

**Table S4. Differentially expressed genes between primary MEFs and SV40-CMEFs.** (Provided as a separate Excel file: Supplementary Table S4. Differentially expressed genes results.xlsx)

**Supplementary Table S5. GSEA results for GO biological process and KEGG pathway collections**. (Please see the separate Excel file: Supplementary Table S5. GSEA results.xlsx)
